# Effect of fit-for-purpose biochars on rumen fermentation, microbial communities, and methane production in cattle

**DOI:** 10.3389/fmicb.2024.1463817

**Published:** 2024-11-19

**Authors:** Gonzalo Martinez-Fernandez, Robert D. Kinley, Wendy J. M. Smith, Jessica Simington, Stephen Joseph, Sara Tahery, Zoey Durmic, Phil Vercoe

**Affiliations:** ^1^Agriculture and Food, CSIRO, St Lucia, QLD, Australia; ^2^Agriculture and Food, CSIRO, Townsville, QLD, Australia; ^3^FutureFeed Pty Ltd, Townsville, QLD, Australia; ^4^School of Materials Science and Engineering, University of New South Wales, Sydney, NSW, Australia; ^5^School of Agriculture and Environment, The University of Western Australia, Perth, WA, Australia; ^6^Institute of Agriculture, The University of Western Australia, Perth, WA, Australia

**Keywords:** biochar, rumen, microbial communities, greenhouse gas, livestock

## Abstract

**Introduction:**

Biochar has gained significant attention as a possible anti-methanogenic supplement for ruminants due to its potential to reduce methane (CH₄) emissions from enteric fermentation. However, its effects on rumen methanogenesis have been inconsistent and, in some cases, contradictory. These variations are likely influenced by factors such as the type of biochar used, its source material, and how it is administered, including the form in which it is provided and the dosage needed to achieve desired outcomes. This study aimed to examine the effects of two fit-for-purpose biochars on rumen fermentation, CH_4_ emissions, and the rumen microbiome of cattle-fed roughage-based diets. Two experiments were conducted to assess the potential of biochar in mitigating CH_4_ emissions.

**Experiment 1:**

This was a controlled pen trial conducted over 56 days, involving 12 steers that were fed Rhodes grass hay *ad libitum*. The animals were assigned to one of four treatment groups: control (no biochar, only molasses), low dose (50 g biochar/animal/day), mid dose (100 g biochar/animal/day), or high dose (200 g biochar/animal/day). Two types of biochar, Biochar 1 and Biochar 2, were administered with molasses (200 mL per animal/day). Methane emissions were measured using open-circuit respiration chambers, and rumen fluid samples were collected for analysis of the rumen microbial community and fermentation metabolite.

**Experiment 2:**

In this trial, 45 heifers were selected and grazed together in a single paddock for 60 days to assess the effects of biochar on productivity and CH_4_ emissions under grazing conditions. The animals were allocated to one of three treatment groups (15 animals per group): control (no biochar, only molasses), Biochar 1, or Biochar 2. Each group was administered biochar at an estimated single dose of 100 g per animal/day mixed with molasses. Methane emissions were measured using GreenFeed systems in the field to monitor CH₄ production from individual animals.

**Results:**

In the controlled pen trial (Experiment 1), biochar supplementation resulted in a reduction of CH₄ emissions by 8.8–12.9% without any negative effects on rumen fermentation or dry matter intake (DMI). Minor changes were observed in the rumen bacterial community, particularly in the *Christensenellaceae* and *Prevotellaceae* families. However, in the grazing trial (Experiment 2), no significant differences in CH₄ emissions or productivity were detected with biochar supplementation.

**Conclusion:**

While the results from controlled feeding conditions suggest that biochar has the potential to reduce enteric CH₄ emissions, the lack of significant findings under grazing conditions highlights the need for further research. Future studies should focus on identifying biochar types, doses, and delivery methods that are effective in reducing CH₄ emissions in grazing systems without compromising cattle productivity.

## 1 Introduction

Methane is the main enteric greenhouse gas emitted from livestock, with a warming potential 28 times greater than carbon dioxide (Gerber et al., 2013), and is estimated to represent between 7 and 18% of total anthropogenic emissions (Hristov et al., 2013). Enteric CH_4_ is an end product of ruminal fermentation and also represents an energy loss of between 2 and 12% of gross energy intake from digested feed for the animal (Johnson and Johnson, 1995; Janssen, 2010). Not surprisingly, there has been a global interest in finding practical solutions to mitigate methane emissions because of the dual benefit of reducing the contribution to global greenhouse gas emissions and the potential to improve production efficiency.

Biochar, a carbon-rich by-product, has been used as a feed additive in livestock since the late 19th century (Totusek and Beeson, 1953). Recently, livestock producers have been feeding biochar to cattle and sheep because there is evidence that soil health might be improved through the distribution of biochar in the soil by dung beetles (Tahery et al., 2023). The thermal conversion of biomass residues to produce a carbon-rich material referred to as charcoal, or, if applied to the ground, biochar, is an accepted method to sequester carbon, reduce emissions from soils and decomposing manures, recycle nutrients, and improve soil health.

Biochar has also gained attention as a possible rumen modifier, particularly in reducing enteric CH_4_ emissions from ruminants (Schmidt et al., 2019). However, the effects of biochar on rumen methanogenesis are variable and often contradictory, with some studies showing no effect (Terry et al., 2019; Winders et al., 2019; Sperber et al., 2022) when feeding biochar to cattle and other studies reporting more than 30% methane decrease in ruminants (Leng et al., 2012; Al-Azzawi et al., 2021). These effects presumably depend on the types of biochar, the parent material source, and the administration regimes in terms of the form it offers and the dosages required to be effective.

Preliminary *in vitro* work has demonstrated that different biochars vary in their anti-methanogenic potential, and biochar production can be manipulated to achieve greater effects (Durmic et al., 2021; Martinez-Fernandez et al., 2022). The properties of biochar can be altered through pre-treatment of the biomass or post-treatment of the biochar. These fit-for-purpose biochars are often referred to as engineered biochars.

The objective of the current study was to examine the effect of supplementing fit-for-purpose biochar to beef cattle on CH_4_ emissions, rumen microbial composition, and fermentation parameters in controlled feeding conditions at different levels and its applicability and effect under grazing conditions.

## 2 Materials and methods

Two experiments were conducted at Lansdown Research Station (Townsville, QLD, Australia). Experiment 1 was a controlled pen trial to select the appropriate dose of biochar for Experiment 2, which evaluated the biochar under grazing conditions. The experimental protocols complied with the Australian Code for the Care and Use of Animals for Scientific Purposes (eighth edition, 2013) and were approved by the CSIRO Animal Experimentation and Ethics Committee (approval no. 2020–13 and 21–06). The 3 R principle was applied to both trials (replacement, reduction, and refinement).

### 2.1 Biochar composition and characterization

A commercial wood biochar (Biochar 1) and a custom-made wheat straw biochar (Biochar 2) that showed the greatest anti-methanogenic effect *in vitro* (Durmic et al., 2021; Martinez-Fernandez et al., 2022) were selected for the *in vivo* experiments (Experiments 1 and 2). The composition and production conditions of the biochars are shown in Table 1.

**Table 1:** Biochar composition, pyrolysis conditions, and manipulation.

Detail	Biochar 1	Biochar 2
Parent material (%)		
*Eucalyptus* spp.	–	28.5
*Acacia cambagei*	100	–
Wheat straw	–	28.5
Wheat straw ash	–	14.3
Zeolite	–	14.3
Bentonite	–	14.3
Pyrolysis temperature (°C)	450	600
Holding time at the highest heating temperature (h)	12	35
Post-pyrolysis manipulation (g/100 g biochar)		
Acidified	–	6 (11 M HCl)
KNO_3_	–	6.6
NaCl	31.5	–
CaCO_3_	30.0	–
Molasses	8.5	–
pH	11.43	4.41

Each biochar’s physical and chemical properties were characterized as follows: pH and electrical conductivity (EC) were measured following the published methodology (Rayment and Lyons, 2010). An Elementar vario MACRO cube combustion analyzer measured total carbon and nitrogen. Scanning electron microscopy (SEM) was used on a FEI NanoSEM 450, attached with a Bruker X-ray energy dispersive spectrometry (EDS) detector, to understand the structural and elemental changes in the samples. Fourier transform infrared spectroscopy (FTIR) was used to determine the functional groups and chemical bonds in the biochars using the technique and instrument setup detailed in Taherymoosavi et al. (2016). The spectrum was obtained over 32 scans and 32 accumulations. Both particle size and zeta potential were measured using a Malvern zeta sizer Nano ZS and the method described by Mukherjee et al. (2011). Zeta potential measures the potential difference between the surface of a solid particle immersed in water. Colloids with high zeta potential (negative or positive) are electrically stabilized, while colloids with low zeta potential tend to coagulate or flocculate. Liquid chromatography—organic carbon detection (LC-OCD) of the filtered solution (0.45 μm filter) to determine the concentration of water-soluble organic carbon and its fractions in the biochars (Taherymoosavi et al., 2016). Soluble elements of the filtrate were measured using inductively coupled plasma mass spectrometry (ICP-MS), and the procedure was detailed in Tahery et al. (2022). Cyclic voltammetry (CV) was used to measure the redox characteristics of the biochars using the procedure detailed in Hossain et al. (2021).

### 2.2 Experiment 1

#### 2.2.1 Experimental design

Twelve steers (*Bos taurus* x *Bos indicus*, mean LW 422 ± 9.9 kg, 3.5 years old) were used in the experiment at Lansdown Research Station (Townsville, QLD, Australia). Animals were randomly allocated to two groups (six animals per group), each group receiving a different biochar (Biochar 1 or Biochar 2). During the trial, animals were fed Rhodes grass hay (*Chloris gayana*) and *ad libitum*. Hay chemical composition: DM 907 g/kg fresh matter; in g/kg of DM: CP, 138; NDF, 688; ADF, 375.

Individual pens were hosed down for cleaning, and the water supply was checked every day before feeding. Fresh feed was provided between 08:00 and 09:00 each day. Each pen contained environmental enhancements of interest to cattle, including a clear vision of the neighboring cohort and suspended toys. Water from an automatic filler, a reticulated water bowl, and feed were always available.

Animals were adapted to the hay for 35 days and then transferred to individual pens for the measurements of individual feed intakes during the experiment. While in individual pens, each group of animals received 200 mL molasses/animal/day (without biochar) for 14 days, with the last 48 h being confined in open-circuit respiration chambers to measure CH_4_ and H_2_ production (control period).

Following the initial control period, each group of animals received a low dose of one of the biochars (50 g biochar 1 or 2/animal/day) mixed with 200 mL molasses/animal/day for 14 days, with the last 48 h spent in open-circuit respiration chambers for direct measurement of CH_4_ and H_2_ production (low dose period). Doses of both biochars were then increased to a mid-level (100 g biochar/animal/day) mixed with 200 mL molasses/animal/day for another 14 days, with the last 48 h placed in open-circuit respiration chambers (mid-dose period). After the mid-dose period, both biochar levels were increased to a high level (200 g biochar/animal/day) mixed with 200 mL molasses/animal/day for 14 days, with the same sampling regime for CH_4_ and H_2_ for the last 48 h.

The biochar doses were selected based on *in vitro* and *in vivo* studies (Schmidt et al., 2019; Durmic et al., 2021; Martinez-Fernandez et al., 2022). Each dose of biochar mixed with molasses was split into two shots and offered daily at 0 and 6 h after feeding the hay.

Rumen fluid and blood samples were collected from the animals at the end of each respiration chamber period. Rumen fluid samples were collected 3 h post-feeding by oesophageal intubation.

Samples were immediately frozen using dry ice and stored at −20°C for ruminal fermentation metabolites or at −80°C for subsequent DNA extractions to study rumen microbial community composition. Blood samples from all animals were collected by jugular venipuncture using a 10 mL blood Vacutainer tube (BD, Sydney, Australia) coated with silica for serum.

Blood samples for serum were kept for 1 h at room temperature before being placed on ice for centrifugation. Blood samples were centrifuged at 2,500 rpm for 20 min at 4°C to separate the serum, which was then stored at −80°C for blood urea nitrogen (BUN) analysis.

#### 2.2.2 Respiration chamber measurements

Four open-circuit respiration chambers were used to determine CH_4_ and H_2_ production from individual steers, as described by Martinez-Fernandez et al. (2016). Briefly, CH_4_ and H_2_ emissions were detected using four clear polycarbonate independent pens with an air volume of 23.04 m^3^ and an airflow of 3,000 L/min and maintained at a negative pressure (− 5.1 ± 0.14 Pa).

Air samples were passed through a chemical drier and were re-metered through independent rotameters before compositional analysis for CH_4_ (Servomex 4,100, Servomex Group Ltd., Crowborough, United Kingdom) and H_2_ (Dräger X-am 5,000, Draeger Safety Pacific Pty. Ltd., Notting Hill, VIC, Australia). CH_4_ and H_2_ production (g) were calculated by averaging individual animal measurements for 48 h.

#### 2.2.3 Analytical methods

Feed samples were dried in a forced-air oven at 105°C to constant weight prior to grinding. Feed samples were ground through a 1 mm sieve before analysis. Dry matter (DM), ash, neutral detergent fiber (NDF), acid detergent fiber (ADF), and total nitrogen contents were analyzed at the CSIRO Floreat laboratory (Floreat, WA, Australia).

Concentrations of volatile fatty acids (VFAs) (acetate, propionate, n-butyrate, iso-butyrate, iso-valerate, and n-valerate) were measured in rumen fluid samples using gas chromatography (GC) as described by Gagen et al. (2014). Iso-valerate (3-methyl butyrate) includes 2-methylbutyrate, which co-elutes.

The NH_3_-N concentration in rumen fluid and blood urea nitrogen (BUN) was determined using the method described by Chaney and Marbach (1962).

#### 2.2.4 Rumen microbial analyses

The DNA extractions from rumen samples were performed as described by Martinez-Fernandez et al. (2016). The 16S rRNA gene was used to characterize the microbial populations in the rumen for bacteria (v4 region) (Kozich et al., 2013). Each DNA sample was amplified using specific primers and a unique barcode combination, as described by de Carcer et al. (2011). Amplification products were visualized by performing gel electrophoresis. Product quantities were calculated, and an equal molar amount of each target product was pooled. The pooled target products were run in a 1.5% agarose gel, and bands were visualized and excised under blue light trans-illumination. The amplicons were gel purified with a QIAquick Gel Extraction Kit (Qiagen, Hilden, Germany) prior to submission for 2 × 300 bp Illumina MiSeq sequencing (Australian Centre for Ecogenomics, University of Queensland). Paired-end short-read sequence data generated on the Illumina MiSeq was processed using the USEARCH package (Edgar, 2010). De-multiplexed paired-end sequences were first merged prior to sequence quality filtering, followed by denoising (error correction), chimera checking, and clustering of sequences to Amplicon sequence variants (ASVs) (Callahan et al., 2017). Analysis of microbiota diversity and identification of ASVs significantly altered by supplementation or dam was performed in R studio following the compositional data analysis (Gloor et al., 2017), using packages mixOmics (Rohart et al., 2017), phyloseq (McMurdie and Holmes, 2013), propr (Quinn et al., 2017), vegan (Oksanen et al., 2019), ALDEx2 (Gloor et al., 2016), and metacoder (Foster et al., 2017). Taxonomic classification of bacterial ASVs was done using the IDTAXA algorithm implemented in the DECIPHER R package against the SILVA SSU r132 training set (Murali et al., 2018).

The DNA samples were also used as templates for quantifying the abundance of the mcrA gene for total methanogens and the 16S rDNA for Methanobrevibacter and Methanomassiliicoccaceae family specific. The primers and assay conditions used were previously published by Denman et al. (2007) and Huang et al. (2016). Real-time PCR (qPCR) analyses were run in quadruplicate from one DNA extraction on an Applied Biosystems™ ViiA™ 7 Real-Time PCR System (Thermo Fisher Scientific Inc.). Assays were set up using the SensiFAST SYBR® Lo-ROX reagents (Bioline). Assay conditions were optimized for primer, template DNA, and MgCl_2_ concentrations.

An optimal primer concentration of 400 nM and a final MgCl_2_ concentration of 3 mM was used for each assay under the following cycle conditions: one cycle of 50°C for 10 s and 95°C for 2 min 30 s for initial denaturation, 40 cycles at 95°C for 15 s and 60°C for 1 min for primer annealing and product elongation. Fluorescence detection was performed at the end of each annealing and extension step. Amplicon specificity was performed via dissociation curve analysis of PCR end products by raising the temperature at a rate of 0.05°C /s from 60 to 95°C. Changes in targeted populations were calculated using a relative quantification calculation and the 2-∆∆Ct method, with the control period used as the calibrator and total bacterial Ct (cycle threshold) values used as the reference value (Livak and Schmittgen, 2001; Denman and McSweeney, 2006).

### 2.3 Experiment 2

#### 2.3.1 Experimental design

A total of 45 heifers (*Bos taurus* x *Bos indicus*, 11 ± 2 months of age, BW 252 ± 57 kg) were selected and grazed for 60 days in the same paddock (~45 ha) at Lansdown Research Station in Northern Australia (Townsville, QLD, Australia). Paddock grasses and legume composition: *Urochloa* sp., Rhodes grass (*Chloris gayana*), Bluegrass (*Dichanthium sericium*), buffel (*Cenchrus ciliaris*) and spear grass (*Heteropogon contortus*); legumes: Seca Stylo (*Stylosanthes scabra*), Verano (*Stylosanthes hamata*) and Desmanthus (*Desmanthus* sp.). Paddock pasture’s average nutrient composition (g/kg DM) was 82 CP, 688 NDF, 411 ADF, 277 hemicellulose, and 66 ash.

Animals were randomly allocated to three groups, each receiving one of the following treatments: Control group: 2,900 mL molasses/group/day. Biochar 1 group: 1.5 kg biochar mixed with 2,900 mL of molasses/group/day, and Biochar 2 group: 1.5 kg biochar mixed with 2,900 mL of molasses/group/day.

The dose used was equivalent to 100 g biochar/animal/day, which was selected based on the results of Experiment 1. The amount offered was also in line with industry recommendations, which suggest administering between 50 and 100 g of biochar/animal/day. The study’s objective was to simulate grazing conditions; therefore, the supplements were offered to the animals as a group, meaning individual intake may have varied during the trial.

Animals were allocated to the treatments and water points (daily) using a walk-over weigher (WOW) with auto drafter (Remote WOW Drafter Prime Satellite, Datamars, Brisbane, Australia), which recognized individual RFIDs. All animals were able to access the supplement at the same time when visiting the enclosed areas for drinking water. The treatment mix was evenly distributed in the troughs to limit variation in intake between animals, and it was completely consumed by the animals daily. Individual animal body weights were measured at the paddock daily by the WOW. Enteric CH_4_, H_2,_ and CO_2_ emissions (g/day) were measured from each individual animal using the Greenfeed Emission Monitors.

The cattle were managed as per typical herd management. Staff checked the paddocks five times per week to ensure that animals had adequate access to water and had no injury or disease.

#### 2.3.2 Greenfeed emission monitor measurements

The animals had access to two Greenfeed Emission Monitors (GEM) units (C-Lock Inc., Rapid City, SD, United States) (Zimmerman and Zimmerman, 2012; Hammond et al., 2016). The GEM units were placed at the paddock to measure daily enteric methane emissions for 2 months. To control the number and duration of methane measurements, GEM provided pellets (Barastoc calm performer, Ridley agriproducts) to each animal with a maximum of 4 feeding sessions/d and a minimum of 5 h between sessions. In each feeding session, the maximum quantity of pellets delivered per animal was 175 g (5 drops of approximately 35 g each with 30 s intervals between drops). If cattle did not remain to receive the five drops in 1 visit, they could make further visits to the GEM in that session until the maximum pellet drops were dispensed. For emission data to be recorded, animals were required to have their heads in the unit for at least 2 min as detected by a proximity sensor. Air filters on the GEMs were changed weekly, and gas sensors were calibrated automatically weekly at night when no cattle were accessing the units. CO2 recoveries were performed monthly during the duration of the trial. Daily emission and production estimates (g CH_4_, H_2,_ and CO_2_/d) were all calculated using the valid data provided by C-Lock to generate emission estimates for individual animals on individual days during the measured period.

### 2.4 Statistical analyses

In Experiment 1, the effect of dose was analyzed for CH_4_ and H_2_ production, dry matter intake (DMI), live weight (LW), ruminal fermentation metabolites, and methanogen abundances for each biochar as a univariate repeated-measures analysis of variance using the GLM procedure of SPSS (IBM, version 21.0), with animals as the experimental unit. Linear, cubic, and quadratic components of the response to incremental doses of each biochar were evaluated using polynomial contrasts. A univariate model using the GLM procedure of SPSS was used to compare both biochars. The treatment was considered the fixed effect with the animal as the experimental unit. Effects were declared significant at a *p*-value of ≤0.05, and *p*-values between 0.05 and 0.10 were considered a trend.

In Experiment 2, data were analyzed as a univariate model using the GLM procedure of SPSS (IBM Corp., version 21.0, Armonk, NY, United States). The treatment was considered the fixed effect with the animal as the experimental unit. The effect of treatment was analyzed for body weight (BW), average daily weight gain (ADWG), CH_4_, CO_2,_ and H_2_ emissions (g/day). Effects were declared significant at *p* < 0.05, and p-values between 0.05 and 0.10 were considered a trend.

## 3 Results

### 3.1 Experiment 1

Methane production and yield (g/day and g/kg DMI Table 2) were significantly lower (*p* < 0.05) in animals receiving both biochars compared with the control period. Biochar 1 decreased CH_4_ yield (g/kg DMI) by approximately 8.8 to 10.0% (*p* < 0.05) and Biochar 2 by approximately 9.5 to 12.9% compared with their respective control periods. Only Biochar 2 showed a linear and cubic effect (*p* < 0.05), indicating a dose-dependent response. No significant differences (*p* > 0.05) in the amount of expelled H_2_ were observed between the control period and each biochar. In addition, BW, DMI, CH_4,_ and H_2_ production were not significantly different between biochars (*p* > 0.05) (data not shown).

**Table 2:** Dose–response of Biochar 1 and 2 on DMI, CH_4,_ and H_2_ production in steers feed Rhodes grass hay.

	Control	Low	Mid	High	SEM	*P-*value	Polynomial contrast^1^
Biochar 1							
DMI (kg)	7.91	8.08	8.03	7.90	0.13	n.s.	n.s.
CH_4_ (g/day)	189^a^	176^bc^	182^ab^	170^c^	5.09	0.007	*C*
H_2_ (g/day)	0.00	0.00	0.00	0.00	0.00	n.s.	n.s.
CH_4_ (g/kg DMI)	23.9^a^	21.8^b^	22.8^ab^	21.5^b^	0.40	0.029	n.s.
Biochar 2							
DMI (kg)	8.54	8.61	8.65	8.47	0.33	n.s.	n.s.
CH_4_ (g/day)	197^a^	180^bc^	188^b^	170^c^	6.89	0.001	*L, C*
H_2_ (g/day)	0.00	0.00	0.00	0.00	0.00	n.s.	n.s.
CH_4_ (g/kg DMI)	23.2^a^	21.0^c^	21.7^b^	20.2^c^	0.86	0.001	*L, C*

^a−c Within a row treatment means without a common superscript differ, p < 0.05.^

^1^Polynomial Contrast: Significant (*p* ≤ 0.05) linear (L) or cubic (C) effects if the response to incremental doses of biochar is estimated by polynomial contrast. n.s.: no significant.

Regarding the fermentation parameters, no significant effects were observed between control periods and biochar doses for the VFA profile, ammonia concentrations, rumen pH, and redox potential (*p* > 0.05). The only significant difference detected was an increase in blood urea nitrogen for the second dose of both biochars compared to the control period (*p* < 0.05) (Tables 3, 4). There were no negative effects on DMI, LWG (data not shown), or rumen fermentation parameters (Tables 2–4) with any of the doses of the biochars (*p* > 0.05).

**Table 3:** Rumen fermentation parameters and blood urea nitrogen (BUN) in steers fed Rhodes grass hay without or with Biochar 1 (Experiment 1).

	Control	Low	Mid	High	SEM	*P-*value	Polynomial contrast^1^
Rumen pH	7.14	7.12	6.93	7.18	0.07	0.335	n.s.
Redox potential (mV)	−292	−290	−304	−237	10.8	0.086	n.s.
BUN (mg/100 mL)	23.9^b^	24.0^b^	27.2^a^	24.6^b^	0.92	0.050	*L, C*
Ammonia-N (mg/100 mL)	12.4	12.9	12.0	11.3	0.18	0.727	n.s.
Total VFA (mM)	62.2	50.7	58.7	57.0	3.56	0.081	n.s.
(mol/100 mol)							
Acetate	74.2	74.5	74.5	75.3	0.44	0.737	n.s.
Propionate	13.6	14.2	14.1	14.1	0.11	0.308	n.s.
Iso-Butyrate	1.38	1.35	1.36	1.43	0.02	0.226	n.s.
N-Butyrate	6.50	6.61	6.85	6.25	0.18	0.133	n.s.
Iso-Valerate	1.45	1.16	1.10	1.16	0.06	0.402	n.s.
N-Valerate	1.37	0.91	1.00	0.86	0.10	0.353	n.s.
N-Caproate	1.46	1.31	1.12	0.95	0.11	0.543	n.s.
Ratio A:P	5.46	5.26	5.30	5.37	0.06	0.538	n.s.

^a−b Within a row treatment means without a common superscript differ, P < 0.05.^

^1^Polynomial Contrast: Significant (*P* ≤ 0.05) linear (L) or cubic (C) effects if the response to incremental doses of biochar is estimated by polynomial contrast. n.s.: no significant.

**Table 4:** Rumen fermentation parameters and blood urea nitrogen (BUN) in steers fed Rhodes grass hay without or with Biochar 2 (Experiment 1).

	Control	Low	Mid	High	SEM	*P-*value	Polynomial contrast^1^
Rumen pH	7.04	6.96	7.21	7.06	0.04	0.452	n.s.
Redox potential (mV)	−298	−283	−301	−267	5.76	0.124	n.s.
BUN (mg/100 mL)	24.5^c^	25.4^b^	27.9^a^	24.0^c^	0.53	0.001	*Q, C*
Ammonia-N (mg/100 mL)	13.8	15.3	14.1	11.4	0.84	0.191	n.s.
Total VFA (mM)	63.1	59.9	48.7	56.2	1.68	0.112	n.s.
(mol/100 mol)							
Acetate	75.8	75.3	75.0	75.5	0.2	0.581	n.s.
Propionate	13.4	13.9	13.5	13.5	0.11	0.718	n.s.
Iso-Butyrate	1.33	1.40	1.49	1.46	0.05	0.069	n.s.
N-Butyrate	6.33	6.20	6.64	6.26	0.2	0.341	n.s.
Iso-Valerate	1.12	1.18	1.19	1.17	0.05	0.719	n.s.
N-Valerate	0.97	1.07	1.03	0.91	0.03	0.095	n.s.
N-Caproate	1.06	1.03	1.12	1.15	0.05	0.878	n.s.
Ratio A:P	5.65	5.45	5.55	5.59	0.05	0.796	n.s.

^a−c Within a row treatment means without a common superscript differ, P < 0.05.^

^1^Polynomial Contrast: Significant (*P* ≤ 0.05) cubic (C) or quadratic (Q) effects on the response to incremental doses of biochar estimated by polynomial contrast. n.s.: no significant.

**Figure 1:**
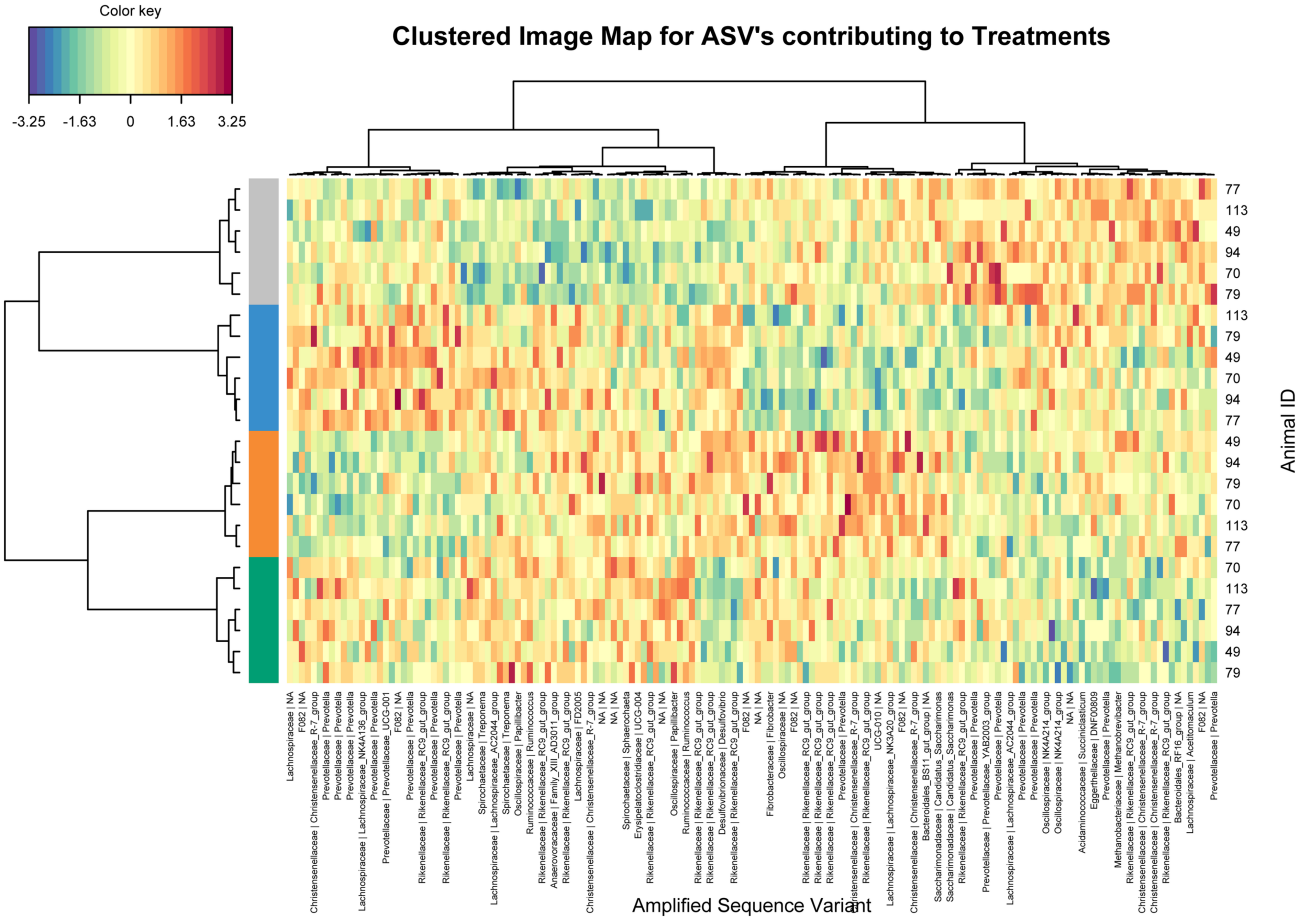
Clustering analysis using a heatmap based on selected bacterial ASVs that best characterized the rumen bacterial profile of the steers supplemented with Biochar 1. Treatments: Blue color control, orange color low dose, grey color mid dose and green color high dose.

**Figure 2:**
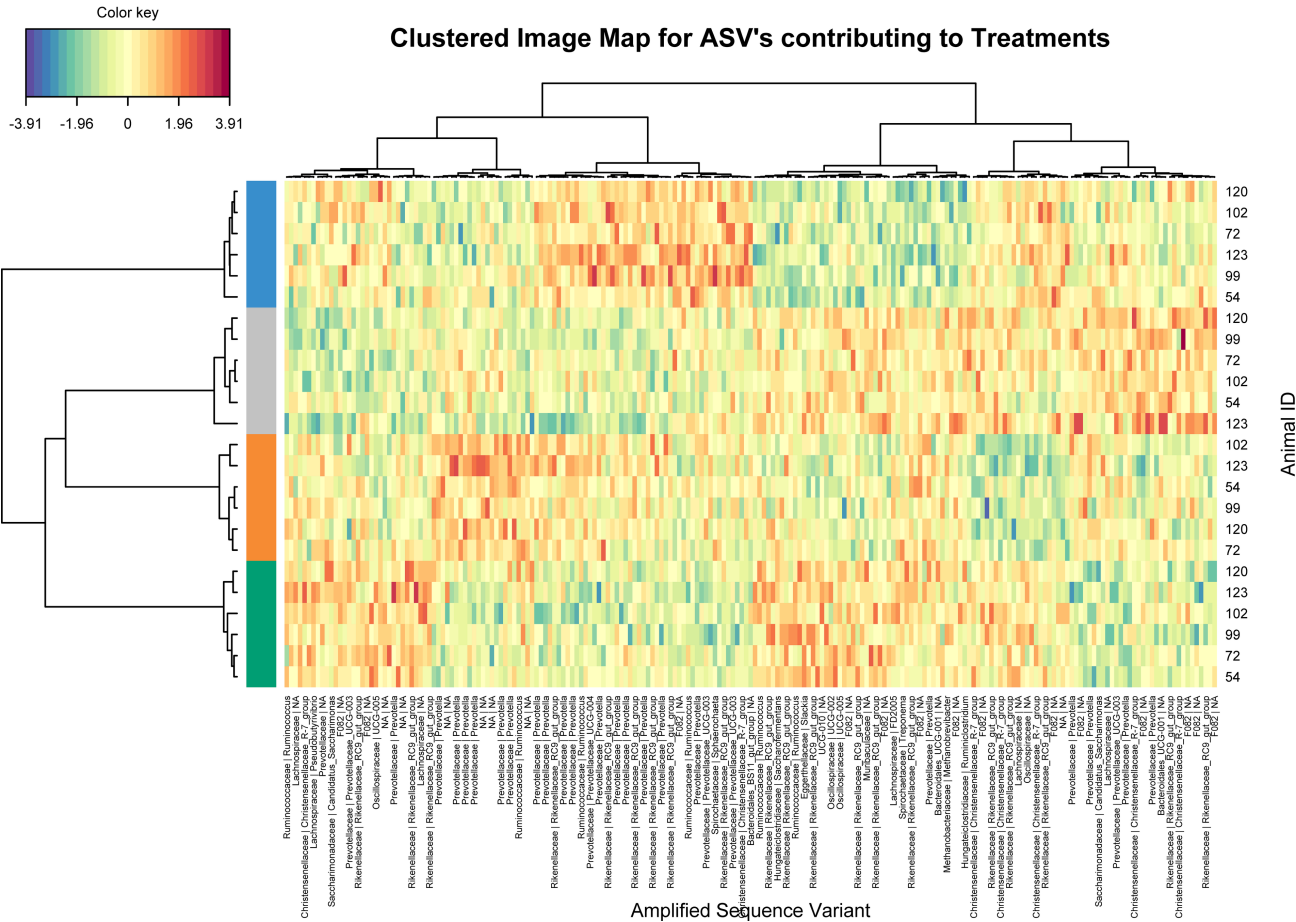
Clustering analysis using a heatmap based on selected bacterial ASVs that best characterized the rumen bacterial profile of the steers supplemented with Biochar 2. Treatments: Blue color control, orange color low dose, grey color mid dose and green color high dose.

Using sparse PLS discriminant analysis (sPLS-DA), ASVs that best characterized the animal treatment groups were determined (Lê Cao et al., 2011). The analysis showed minor differences in rumen bacterial structure between supplemented and unsupplemented animals (Supplementary Figure S1). Clustered image heatmaps (mixOmics analysis) for the selected rumen bacterial ASVs at control and three levels of each biochar showed a distinct microbial signature for both biochars compared to control. The selected ASVs based on the sPLS-DA analysis are visualized using a differential taxonomic heat tree (Figures 1, 2). The main bacterial ASV positively associated with Biochar 1 (Figure 1) is classified into the families *Rikenellaceae*, *Christensenellaceae,* and *Prevotellaceae* for the three doses and the genera *Candidatus Saccharimonas* for the low and mid doses. The main bacterial ASV positively associated with Biochar 2 (Figure 2) is classified into the family *Prevotellaceae* (particularly for the lowest dose), *Christensenellaceae*, *Rikenellaceae,* and *Ruminococcaceae*. No significant differences in abundances of methanogens were observed when biochars were fed to the animals compared to the control (Supplementary Figure S2).

### 3.2 Experiment 2

Measurements from the GreenFeed units that passed quality control totaled 1,687 for the 2-month trial, with an average of 562 visits per treatment group and 46 visits per animal. The CH_4_, CO_2,_ and H_2_ production (g/day) did not significantly differ between the control and the animals supplemented with the biochar. No significant differences in BW and ADWG were detected between control and biochar-supplemented animals (Table 5).

**Table 5:** Biochar effects on body weight, ADWG, CH_4_, H_2,_ and CO_2_ production in cattle grazing Northern Australia tropical forage (Experiment 2).

	Control	Biochar 1	Biochar 2	SEM	*P-*value
BW (kg) prior to supplementation	251	251	254	8.72	0.983
BW (kg) end supplementation	265	262	265	7.69	0.982
ADWG (kg)	0.161	0.134	0.127	0.02	0.711
CH_4_ (g/day)	181	180	185	6.66	0.866
H_2_ (g/day)	0.54	0.54	0.51	0.07	0.944
CO_2_ (g/day)	4,181	4,007	4,190	171	0.711

### 3.3 Biochars characterization

The physical and chemical properties of both biochars used in the animal trials were characterized. Biochar 1 had a higher pH (9.73), acid neutralizing capacity (24.34% CaCO_3_), and EC (50.27 dS/m) than Biochar 2 (pH 4.91, 0.24% CaCO_3_, and 10.22 dS/m) but a lower moisture content (6.7% vs. 25.5%), total organic carbon (13.7% vs. 24.2%), BET surface area (0.1427 m^2^/g vs. 6.505 m^2^/g), and ash content (52.6% vs. 60.2%) (Supplementary Table S1). Biochar 1 had a higher content of Na, Ca, S, and Zn but a lower content of Fe, Si, P, Mn, and Al (Supplementary Table S2). Polychlorinated biphenyls (PCB) and polycyclic aromatic hydrocarbons (PAH) were not detected in the biochars.

Analysis of the surface (Supplementary Table S3) indicated that the concentration of total surface C functionalities was higher in Biochar 1 (52.63%) than in Biochar 2 (20.48%). The main surface C functional group was C-C/C-H/C=C at 284.8 eV, which was higher in Biochar 1 (34.01%). In addition, the relative portions of C-O (at 286 eV) and carboxylic functional group (at 289.2 eV) were greater in Biochar 1. However, Biochar 2 showed a higher total surface N functionality (2.45%), with nitrate (at 407 eV) and N-O in nitrite and/or chemisorbed NH_3_ only detected in Biochar 2. Similarly, Fe functional groups, including Fe_2_(SO_4_)_2_ and Fe^3+^, and Mg, Al, and Si functionalities were only detected in the Biochar 2. This is consistent with the elemental analysis (Supplementary Table S2), where a significantly higher Fe was found in the Biochar 2. No S functionalities were found on the surface of the Biochar 2. A higher surface Na functional group was found in Biochar 1 (6.17%), which is also supported by the elemental analysis (Supplementary Table S2). Quantitative analysis of the DOC fractions (Supplementary Table S4) showed a higher concentration of DOC in Biochar 1 (15.41 mg/g). The majority of the dissolved organic carbon fraction in both biochars were low molecular weight neutrals and humic-like substances (Supplementary Table S4). Biochar 1 had a higher negative zeta potential (−12.5 mV) than Biochar 2 (−3.4) (Supplementary Table S5).

Two wavenumber regions are shown in the FTIR spectra (Supplementary Figure S3): the peaks between 4,000 and 2,600 cm^−1^ and those between 1,800 and 450 cm^−1^. These functional groups play a crucial role in determining the ability of biochar to bind with different nutrients when ingested by the animal. The broad peak between 3,400 cm^−1^ and 3,200 cm^−1^ can be attributed to O-H (alcohol or phenol) and aromatic C-H stretch (alkenes). This broad peak was less intense in Biochar 2 and appeared as a single peak in Biochar 1 (approximately 3,390 cm^−1^).

Additionally, two sharp peaks, approximately 3,000 cm^−1^, likely corresponding to either C-H stretch in alkanes or O-H in carboxylic acids, and at 2800 cm^−1^, were only found in Biochar 2. The intense peak near 1,415 cm^−1^ in Biochar 1 is associated with C-H groups in alkanes, aldehydes, and ketones and may also relate to C-O-H bending in carboxylic acids (which aligns with XPS analysis in Supplementary Table S3). The peak around 1,020 cm^−1^ (and at 1120 cm^−1^ in Biochar 1) could indicate the presence of organic and inorganic compounds such as phosphate, silicate, or Al-, O- and/or C-, O-bonds, with Biochar 2 displaying a higher peak intensity. Small peaks in Biochar 2 (between 800 and 600 cm^−1^) suggest the presence of Al-O and Si-O bonds. Furthermore, Biochar 2 exhibited a slightly higher specific capacitance than Biochar 1 (0.416\u00B0F/g vs. 0.317\u00B0F/g) (Supplementary Figure S4).

## 4 Discussion

The current study was developed to validate *in vivo* the findings of preliminary *in vitro* work where the concept of developing fit-for-purpose biochar products for ruminants was evaluated (Durmic et al., 2021; Martinez-Fernandez et al., 2022).

In the first experiment, the two biochars that demonstrated greater CH_4_ inhibition *in vitro* (Durmic et al., 2021; Martinez-Fernandez et al., 2022) reduced enteric CH_4_ production by 8.8–12.9% when tested in cattle as a supplement under controlled feeding conditions. However, when the same biochars were supplemented to cattle under grazing conditions with voluntary supplement intake in Experiment 2, it did not induce significant differences in enteric CH_4_ emissions. The effect in both scenarios occurred without affecting ADWG or fermentation but induced some changes in ruminal microbial populations compared to the control group under controlled feeding conditions. The present research further supports the concept that not all biochars have anti-methanogenic properties, and variable feeding systems can be expected to impact efficacy. It should be noted that various other factors such as diet, age, and physiology status may influence the outcome and that the present study only tested biochar’s ability to reduce methane in animals consuming low-quality tropical forage. Further trials need to be undertaken to determine if feeding biochar to animals on different diets would have a substantially different outcome.

Published *in vivo* studies describing the biochar effect on enteric CH_4_ production in ruminants are variable and often contradictory, which is likely due to differences in the properties of the biochars used, parent material source, dose, basal diet, and compounds that those specific biochars contained, as well as the class and physiological stage of the animal. For example, Terry et al. (2019) and Sperber et al. (2022) found no significant effect of pine-enhanced biochar on CH_4_ production in cattle fed a barley-silage diet or growing and finishing diets. Similarly, Winders et al. (2019) did not find any inhibition in CH_4_ production in steers on finishing diets (feedlot) but reported a 10% CH_4_ reduction in steers fed growing diets when 0.8% biochar was supplemented. In contrast, Leng et al. (2012) reported up to 24% reduction in CH_4_ concentration when 0.6% biochar was added to the diet of Laos yellow cattle and 40% reduction when biochar was combined with 6% potassium nitrate. However, the CH_4_ measurement techniques used by Leng et al. (2012) were a modified plastic film portable accumulation chamber (PAC) accompanied by short and potentially inconsistent sampling periods and timings of manual air samples and testing, which was not able to measure the methane production during a 24 h period. That said, with the potential for loss of airtight seal and representative sampling leading to inaccuracies, such techniques offer a useful indicator only and are not adequate to accurately quantify the methane production and the extent of methane reduction during a 24-h period. Although promising, further studies are recommended to confirm their biochar’s CH_4_ inhibition using standard techniques.

Similarly, Al-Azzawi et al. (2021) studied the effect of 0.5% DMI of a high-activity microporous powdered activated carbon biochar as a feed supplement in dairy cows and reported a 30–40% reduction in CH_4_ concentration in the circumambient air. Although the technique differed from the study by Leng et al. (2012), the methodology used to measure the CH_4_ emissions, collecting samples from bulk exhaust air in the open-sided barn, is limited to specific time points, as it cannot measure the overall methane production. Again, the results are promising for the product, and the measurement technologies need rigorous testing to confirm accuracy and inhibition claims.

The mechanism by which biochar might inhibit CH_4_ production in rumen has not been identified. Some studies (Leng et al., 2012; Leng et al., 2013; Leng, 2014) have suggested several theories: that biochar might capture CH_4_ gas in the rumen; biochar surface might improve microbial biofilm formation in the rumen; biochar may increase the abundance of methanotrophs; or some particular biochars might contain anti-methanogenic compounds that are released in the rumen. However, these hypotheses still need to be confirmed. The absorption of CH_4_ in the rumen by the biochar seems unlikely due to the variable or lack of effect on enteric methane production of biochar with similar physical properties (Durmic et al., 2021; Martinez-Fernandez et al., 2022). Regarding the methanotrophs, recently Zhang et al. (2019) showed that some biochars can stimulate anaerobic oxidation of CH_4_ by anaerobic methanotrophic archaea in sediments. However, this has not been studied in the anaerobic rumen environment, and the existence of methanotrophs within the rumen has not been consistently proven, with some studies not detecting methanotrophs (Henderson et al., 2015) or being detected at extremely low abundance (Mitsumori et al., 2002; Parmar et al., 2015).

In our study, the characterization of the biochar offered some insight into ancillary features resulting from the parent material and post-pyrolysis modifications, such as the larger particle size in Biochar 1 or the addition of nitrates to Biochar 2, that may have contributed to the biochar effects on rumen fermentation and CH_4_ inhibition under these conditions. The CH_4_ inhibition observed might be produced by indirect inhibition of methanogenesis rather than direct action on the methanogens, based on the compounds identified on the biochars tested, such as nitrates, and the lack of effect on methanogen relative abundances. Nitrate has been evaluated as a feed supplement for reducing enteric methane emissions in livestock with variable results, and caution was conveyed due to increased levels of blood methemoglobin. However, it appears safe under controlled intake scenarios (Tomkins et al., 2018). In the rumen, it acts as an alternative hydrogen sink, redirecting hydrogen from methanogenesis and decreasing the number of rumen protozoa, which results in CH_4_ inhibition (Feng et al., 2020; Ku-Vera et al., 2020). Other biochar characteristics that might affect methanogenesis are discussed below, although they have not been confirmed in the rumen.

Research work in other environments has recently shown that adding MgO to the surface of biochars may absorb and oxidize hydrogen and thus inhibit methane production (Chen et al., 2023). Similarly, MgO can also adsorb methane and CO_2_, which could also inhibit methane production (Manae et al., 2022). Biochar modified with MgO has been found to significantly inhibit methane formation (Ding et al., 2023) in manure. Further research is required to determine if the biochar surfaces can be modified to adsorb and oxidize methane and hydrogen in the rumen. Biochar 1 showed a higher concentration of dissolved organic carbon, the majority of which had a low molecular weight. These can be involved in redox chemistry in an acid environment (Zhou et al., 2014). Low molecular weight organic fractions, which could be a signature of high-temperature biochars (Taherymoosavi et al., 2018), have been reported to assist with transporting nutrients due to their excellent mobility (Tahery et al., 2023). On the other hand, humic-like substances, such as humic acid, fulvic acid, and humin, can improve nutrient uptake by bonding to ions such as Mg, Ca, and Fe (Taherymoosavi et al., 2018). The higher capacitance of Biochar 1 indicates a higher potential to store and donate electrons (Tahery et al., 2022) once the biochar is offered as a feed supplement.

Although both biochars modified the structure of the rumen bacterial populations, it was minor and did not translate into significant changes in rumen fermentation metabolites. Some of the rumen bacterial populations positively associated with the biochars tested, such as C*. Saccharimonas* and *Prevotellaceae,* are propionate producers (Tong et al., 2018). The *Prevotellaceae* family contains a significant representation of genes linked to the propionate pathway (Purushe et al., 2010; Denman et al., 2015). Microbial metabolic processes favoring propionogenesis have been commonly reported when CH_4_ is significantly inhibited (Honan et al., 2022). Although no effect was detected in the current study on rumen fermentation metabolites, the positive effect on microbial populations involved in this metabolic process might suggest that methane inhibition influenced this microbial pathway.

The lower CH_4_ reduction observed in the first *in vivo* trial as compared with short-term and long-term *in vitro* experiments (8.8–12.9% vs. 23–33% reduction) (Durmic et al., 2021; Martinez-Fernandez et al., 2022) has been reported with other antimethanogenic compounds. The direct extrapolation of doses from *in vitro* to *in vivo* systems represents a challenge due to several factors, such as the complexity of the rumen microbial community and digestive physiology, including the rumen fluid passage rate in the animal, compared with the *in vitro* systems (Soto et al., 2012). Martinez-Fernandez et al. (2013, 2015) reported a 33 and 48% CH_4_ reduction when an organosulfur compound was tested in short- and long-term *in vitro* incubations. However, when the same compound and doses were tested in small ruminants (Martinez-Fernandez et al., 2014), they did not find a significant reduction in enteric CH_4_ production, and only a 10% numerical reduction was reported. Another explanation for the lower methane reduction detected *in vivo* compared to the *in vitro* result could be related to the variability inherent in upscaling the manufacturing process of the biochars specifically produced for the *in vivo* trials compared to the ones manufactured at a small scale in the lab for the *in vitro* experiments. Nevertheless, a deeper understanding of the processes inducing the indirect CH_4_ inhibition and how it can be expressed consistently is essential to developing a commercially viable product, particularly for support of CH_4_ reduction claims.

Unexpectedly, no effect on methane was observed when biochars were fed to cattle under grazing conditions in Experiment 2 compared to controlled feeding conditions in Experiment 1. This contrast could be explained by the greater impact and variability of feed consistency, herd, overall DMI, differences in diet selection amongst animals, biochar intake affecting the biochar-feed ratio, and management in extensive grazing conditions vs. intensive livestock systems. That said, Experiment 1 employed respiration chambers for emissions monitoring in contrast to Experiment 2 using GreenFeed monitors. A direct comparison between these methods in a targeted study would be valuable to confirm their compatibility for comparisons between studies.

An effective biochar would require targeted and likely greater enteric CH_4_ reduction capability to achieve a sufficient level of efficacy and be suitable as a cost-effective anti-methanogenic supplement in grazing systems. As demonstrated in this study, controlled feeding results in a more consistent intake of the anti-methanogenic feed additive, which results in improved outcomes compared to grazing systems with voluntary or semi-voluntary intake of the additive. It is inherently challenging for grazing systems to deliver feed additives consistently at effective levels. The uptake and effectiveness of any anti-methanogenic supplement are greater in production systems where feed delivery is more controlled, such as total mixed rations (TMR) typical of feedlots and supplemented systems found in grazing dairy operations. However, a high proportion of enteric CH_4_ is produced worldwide from animals in extensive grazing systems (Roques et al., 2024). Thus, developing new delivery technologies for anti-methanogenic supplements is critical for delivering significant CH_4_ abatement in these systems.

A key element in the adoption of CH_4_ abatement feed additives is the supporting knowledge for carbon crediting schemes. Thus, product delivery technologies for grazing systems must be able to substantiate claims of CH_4_ reduction. While the reduction in CH_4_ observed in the current study was relatively modest, it remains promising. It suggests that further work may optimize biochar, dosage, and conditions to achieve greater methane reductions.

In conclusion, the results of the present study support the notion that not all biochars possess anti-methanogenic properties, and feeding systems can significantly influence their efficacy. The two biochars and doses tested reduced CH_4_ production per g/kg DMI (by 8.8–12.9%) in steers under controlled feeding conditions without adversely affecting rumen fermentation or DMI. However, under grazing conditions, no significant reduction in enteric CH_4_ emissions or ADWG was observed when the same biochars were supplemented over a 60-d period. Consequently, the biochar and doses tested in this study are not suitable as a CH_4_ abatement strategy for grazing cattle. Further research is needed to identify biochar types, doses, and delivery methods that can provide sustained CH_4_ mitigation in grazing systems. To be effective in grazing conditions, supplements will need to have a higher CH_4_ reduction potential and longer efficacy in the rumen to counterbalance the variables in grazing systems that may dilute or diminish the effectiveness observed under controlled feeding conditions.

## Data Availability

The data presented in the study are deposited in the European Nucleotide Archive repository, accession number PRJEB80312 and all the metadata is presented in the main manuscript and additional supporting files.
