# Supplementary material for: Effect of fit-for-purpose biochars on rumen fermentation, microbial communities, and methane production in cattle
Source: Front Microbiol. 2024 Nov 19;15:1463817. doi: 10.3389/fmicb.2024.1463817 (PMC11611548; doi:10.3389/fmicb.2024.1463817)
Supplement: Supplementary file 1 [file Data_Sheet_1.docx]

**Supplementary Table S1.** Basic characteristics of the biochars produced for the animal trials

| Parameter | Biochar 1 | Biochar 2 |
| --- | --- | --- |
| Bulk Density (kg/L) | 0.82 | 0.76 |
| Moisture Content (%) | 6.7 | 25.5 |
| Volatile Matter (%) | 47.4 | 39.8 |
| Ash Content (% ash) | 52.6 | 60.2 |
| pH | 9.73 | 4.91 |
| Electrical Conductivity (dS/m) | 50.27 | 10.22 |
| Sulfur (%) | 1.08 | 0.32 |
| Hydrogen (%) | 1.50 | 1.71 |
| Oxygen (%) | 24.16 | 12.85 |
| Organic Carbon (%) | 13.7 | 24.2 |
| Carbon (%) | 20.5 | 23.8 |
| Nitrogen (%) | 0.17 | 1.04 |
| Acid Neutralising Capacity (% CaCO_3_) | 24.34 | 0.24 |
| Surface Area |  |  |
| Single Point Surface Area P/PO (m^2^/g) | 0.1359 | 6.4509 |
| BET^1^ Surface Area (m^2^/g) | 0.1427 | 6.5050 |
| Langmuir Surface Area (m^2^/g) | 0.1884 | 8.5971 |

^1^ BET: Brunauer, Emmett and Teller

**Supplementary Table S2.** Mineral composition of the biochars**.**

|  | Biochar 1 | Biochar 2 |
| --- | --- | --- |
| % |  |  |
| Ca | 17.03 | 1.16 |
| Mg | 0.17 | 0.24 |
| K | 0.29 | 2.89 |
| Na | 13.29 | 0.54 |
| S | 1.08 | 0.32 |
| P | 0.02 | 0.11 |
| (mg/kg) |  |  |
| Zn | 167 | 31 |
| Mn | 57 | 98 |
| Fe | 849 | 7,080 |
| Cu | 253 | 6 |
| B | <5 | 8.3 |
| Si | 528 | 698 |
| Al | 615 | 9,496 |
| Mo | <1 | <1 |
| Co | 33.9 | 2.40 |
| Se | 7.59 | <1 |
| Cd | 0.5 | <0.5 |
| Pb | <1 | 5.2 |
| As | <2 | 2.48 |
| Cr | 3.2 | 6.7 |
| Ni | <1 | 5.0 |
| Hg | <0.1 | <0.1 |
| Ag | <1 | <1 |

**Supplementary Table S3.** Analysis of the functional groups on the surface of the biochars, measured by XPS.

| Peak name | Functional groups | Binding energy (eV) | Biochar 1 (%) | Biochar 2 (%) |
| --- | --- | --- | --- | --- |
| C1s A | C-C/C-H/C=C | 284.8 | 34.01 | 14.24 |
| C1s B | C-O/C-OC | 286.0 | 10.59 | 3.6 |
| C1s C | C=O | 287.8 | 2.38 | 1.55 |
| C1s D | O=C-O/Carboxylic | 289.2 | 5.65 | 1.09 |
| O1s A |  | 532.02 | 13.94 | 54.55 |
| O1s B |  | 533.34 | 9.05 |  |
| N1s A | N-C-COOH /Pyridone | 400.1 | 0.65 | 0.26 |
| N1s B | Nitrate | 407.69 |  | 1.97 |
| N1s C | Pyridine/N-O/Chemisorbed NH_3_ | 403.88 |  | 0.22 |
| K_2_p_3_ A |  | 293.73 | 0.63 | 2.77 |
| Ca_2_p_3_ A |  | 347.92 | 5.53 | 0.95 |
| S_2_p_3_ A |  | 163.97 | 0.54 |  |
| S_2_p_3_ B |  | 162.05 | 0.14 |  |
| S_2_p_3_ C | Thiosulphate | 168.84 | 0.16 |  |
| Cl_2_p_3_ A |  | 199.42 | 10.55 | 1.35 |
| Na1s |  | 1072.41 | 6.17 | 0.58 |
| Fe_2_p_3_ A | Fe_2_(SO_4_)_2_ | 712.77 |  | 0.43 |
| Fe_2_p_3_ B | Fe3+ | 715.31 |  | 0.13 |
| Si_2_p A |  | 103.4 |  | 9.95 |
| Al_2_s A |  | 120 |  | 4.27 |
| Mg_1_s A |  | 1304.42 |  | 2.08 |

**Supplementary Table S4.** Analysis of the dissolved organic carbon and its fractions, measured by LC-OCD.

| mg/g | Biochar 1 | Biochar 2 |
| --- | --- | --- |
| DOC^1^ | 15.41 | 0.08 |
| Hydrophobic DOC | 3.6 | 0.024 |
| Hydrophilic DOC | 11.82 | 0.054 |
| Biopolymers | 0.11 | 0.001 |
| Humic-like substances | 2.6 | 0.013 |
| Building blocks (polyphenols) | 1.37 | 0.005 |
| LMW^2^ neutrals | 7.73 | 0.034 |
| LMW acids | n.q. | n.q. |

^1^DOC: Dissolved organic carbon, ^2^ LMW: Low molecular weight.

**Supplementary Table S5.** Zeta potential and particle size of the biochars.

| Sample | Particle size (nm) | Zeta potential (mV) |
| --- | --- | --- |
| Biochar 1 | 389.5 | -12.5 |
| Biochar 2 | 156.6 | -3.4 |

**A)**


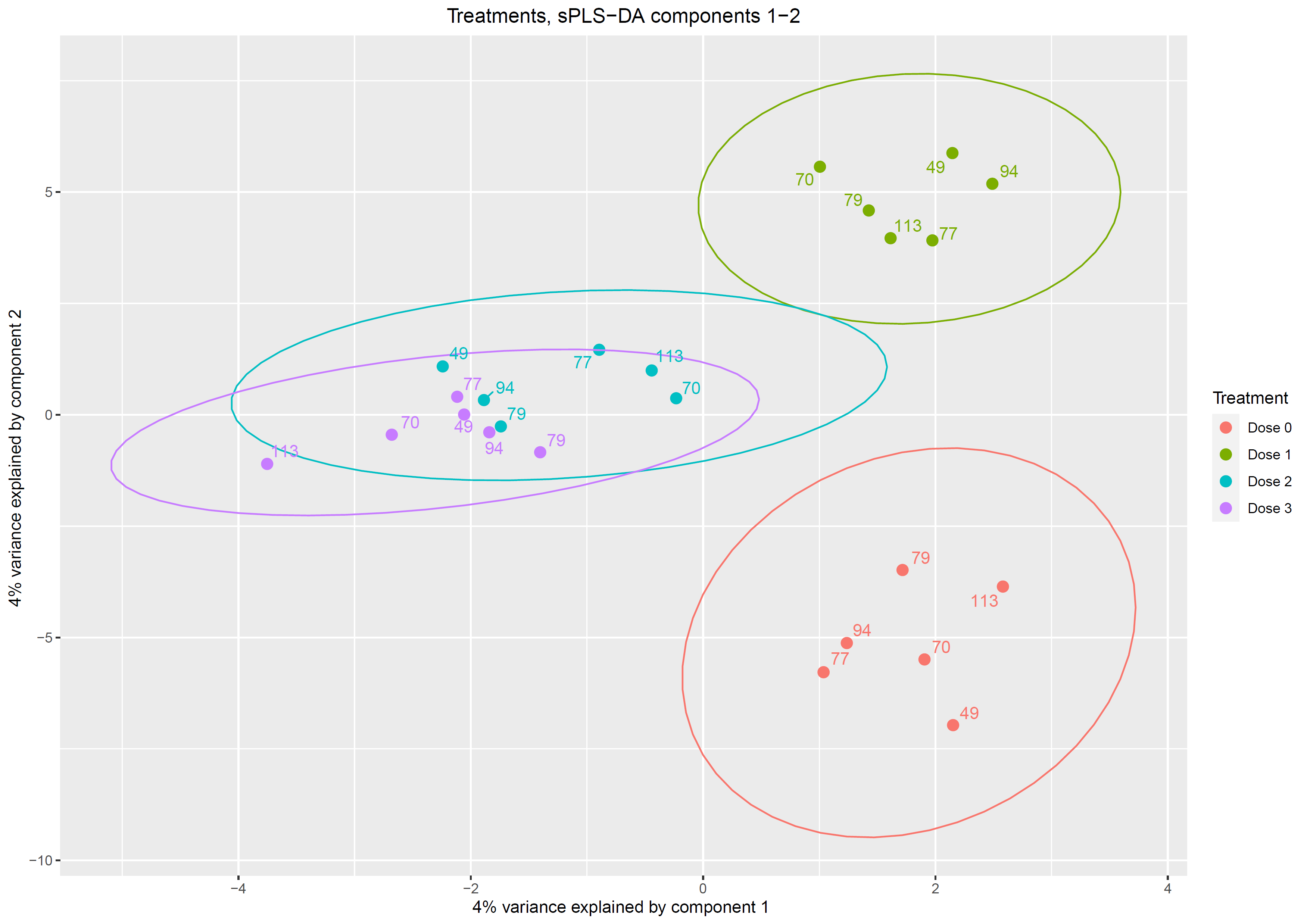


**B)**


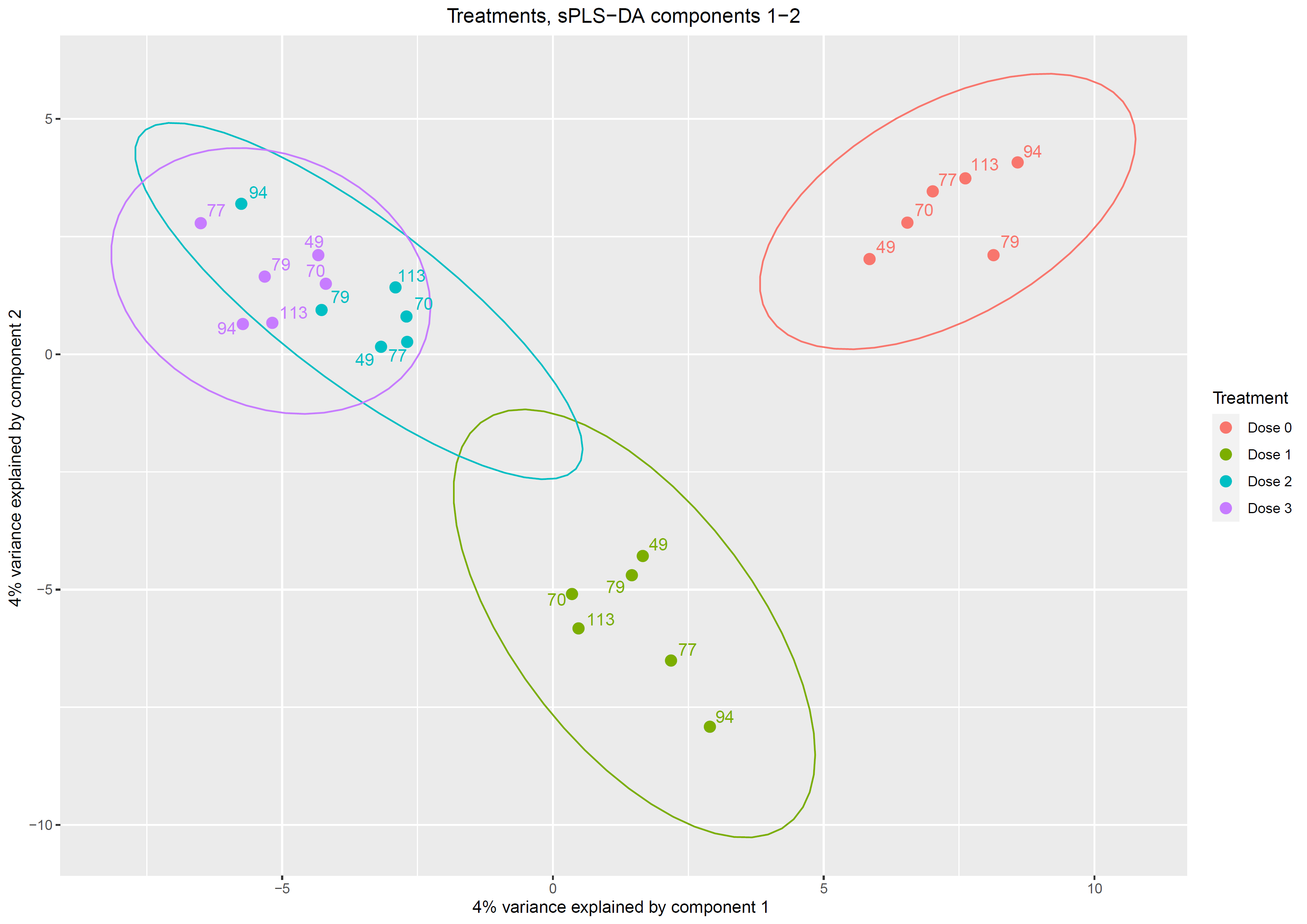


**Supplementary Figure S1.** Supervised analysis with sPLS-DA on rumen bacteria community for cattle supplemented with Biochar 1 (A) and Biochar 2 (B)

**A)**


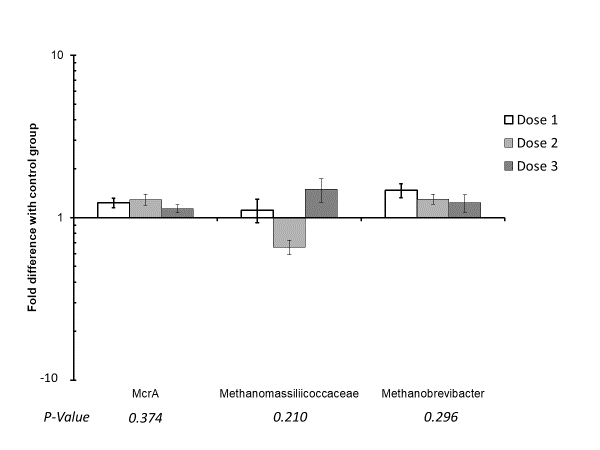


**B)**

**
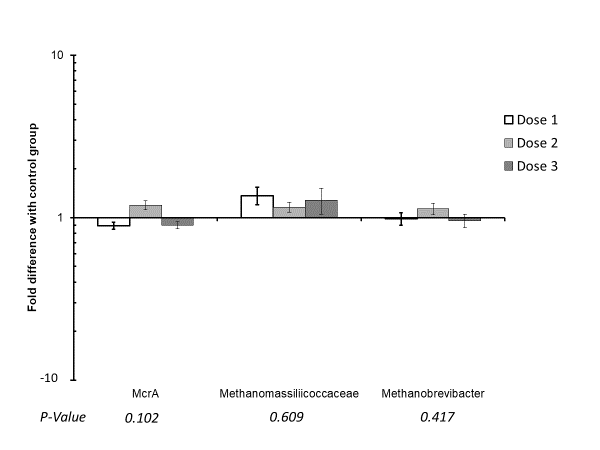
**

**Supplementary Figure S2.** Quantitative PCR (qPCR) analysis of mcrA gene (total methanogens), *Methanobrevibacter* spp. and *Methanomassiliicoccaceae* family population changes in response to the three doses of Biochar 1 (A) and Biochar 2 (B). The y-axis denotes fold change from control period.


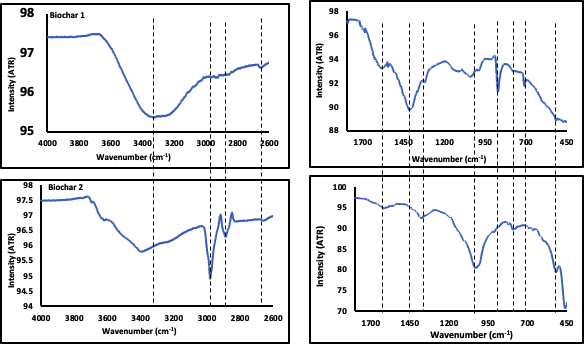


**Supplementary Figure S3.** FTIR spectra of the biochars.

**Supplementary Figure S4.** Cyclic Voltammetry to determine the relative capacitance of the 2 biochars
